# Constitutional mismatch repair deficiency in childhood colorectal cancer harboring a de novo variant in the *MSH6* gene: a case report

**DOI:** 10.1186/s12876-021-01646-3

**Published:** 2021-02-10

**Authors:** Keinosuke Hizuka, Shin-ichiro Hagiwara, Takatoshi Maeyama, Hitoshi Honma, Masanobu Kawai, Kiwamu Akagi, Michiko Yasuhara, Naohiro Tomita, Yuri Etani

**Affiliations:** 1 Department of Gastroenterology, Nutrition and Endocrinology, Osaka Women’s and Children’s Hospital, 840 Murodo-cho, Izumi, Osaka 594-1101 Japan; 2 Division of Molecular Diagnosis and Cancer Prevention, Saitama Cancer Center, 780, Komuro, Ina-machi, Kitaadachi-gun, Saitama 780362-0806 Japan; 3 Division of Lower GI Surgery, Department of Surgery, Hyogo College of Medicine, 1-1 Mukogawa-cho, Nishinomiya, Hyogo 663-8501 Japan; 4 Cancer Treatment Center, Toyonaka Municipal Hospital, 4-14-1, Shibahara-cho, Toyonaka, Osaka 560-8565 Japan

**Keywords:** Café-au-lait macules, Case report, Constitutional mismatch repair deficiency, Mismatch repair gene, Neurofibromatosis type1

## Abstract

**Background:**

Constitutional mismatch repair deficiency (CMMRD) is caused by biallelic pathogenic variants in one of the mismatch repair genes, and results in early onset colorectal cancer, leukemia, brain tumors and other childhood malignancies. Here we report a case of CMMRD with compound heterozygous variants in the *MSH6* gene, including a de novo variant in multiple colorectal cancers.

**Case presentation:**

An 11-year-old girl, who presented with multiple spots resembling café-au-lait macules since birth, developed abdominal pain, diarrhea and bloody stool over two months. Colonoscopy revealed multiple colonic polyps, including a large epithelial tumor, and pathological examination revealed tubular adenocarcinoma. Brain magnetic resonance imaging (MRI) showed an unidentified bright object (UBO), commonly seen in neurofibromatosis type 1 (NF1). Genetic testing revealed compound heterozygous variants, c. [2969T > A (p.Leu990*)] and [3064G > T (p.Glu1022*)] in the *MSH6* gene; c.2969T > A (p.Leu990*) was identified as a de novo variant.

**Conclusions:**

We present the first report of a CMMRD patient with a de novo variant in *MSH6*, who developed colorectal cancer in childhood. CMMRD symptoms often resemble NF1, as observed here. Physicians should become familiar with CMMRD clinical phenotypes for the screening and early detection of cancer.

## Background

Constitutional mismatch repair deficiency (CMMRD; Online Mendelian Inheritance in Man; #276300) is a childhood cancer predisposition syndrome caused by biallelic pathogenic variants in one of four mismatch repair (MMR) genes, i.e., *MLH1, MSH2, MSH6* and *PMS2* [1]. MMR genes recognize and repair mismatched nucleotides, insertions, and deletions during DNA replication. MMR deficiency facilitates the accumulation of genetic changes and consequently, cancer development [2]. From the literature, few case reports have outlined de novo variants in CMMRD. Here, we report a patient with CMMRD who developed multiple colorectal cancers in childhood. The cancer was characterized by compound heterozygous variants in *MSH6*, including a de novo variant.

## Case presentation

**Fig. 1:**
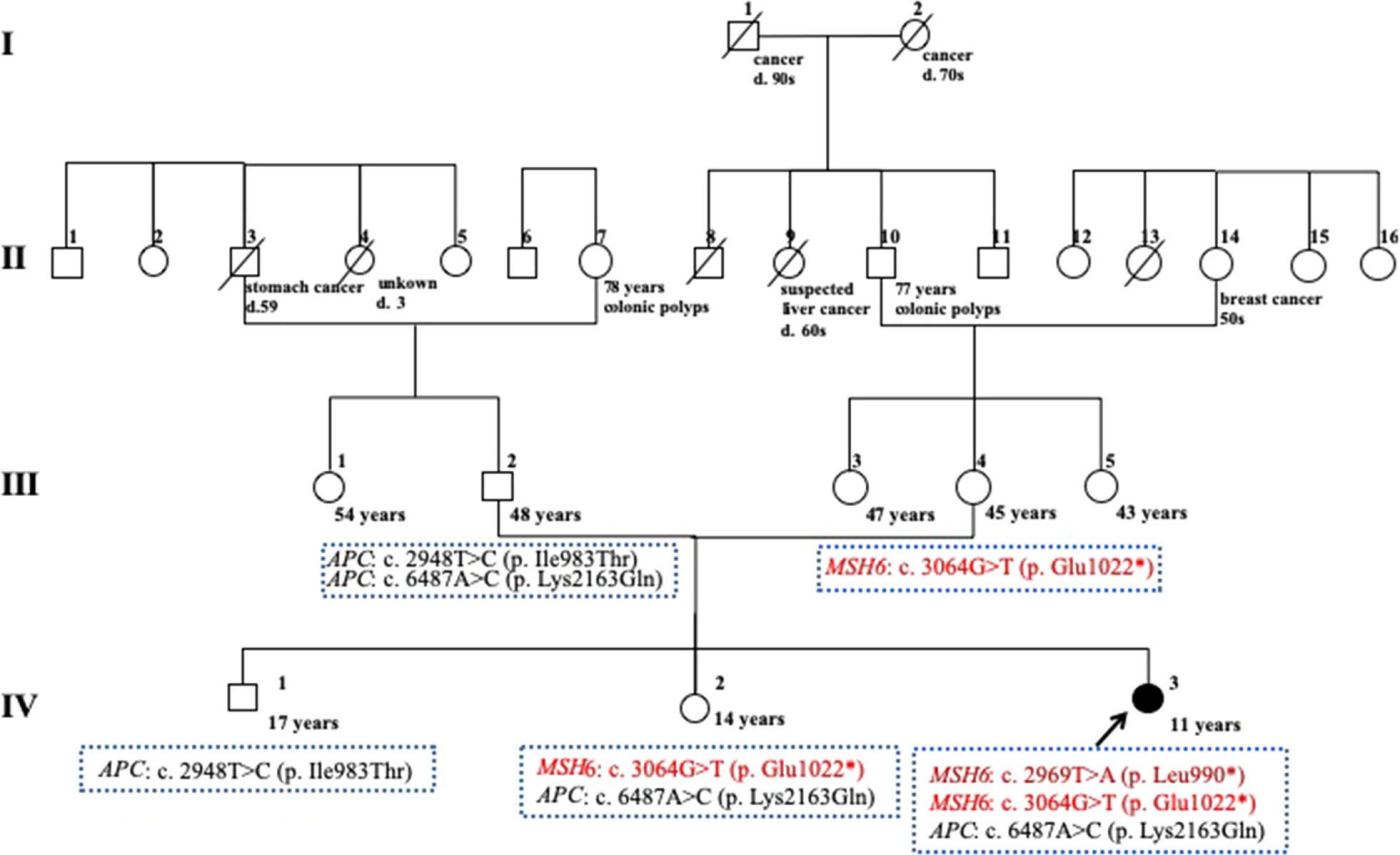
Family pedigree with known medical history. Ages represent (i) age of death for deceased subjects or (ii) the age at the time of medical history collection (10/2018) for living family members

**Fig. 2:**
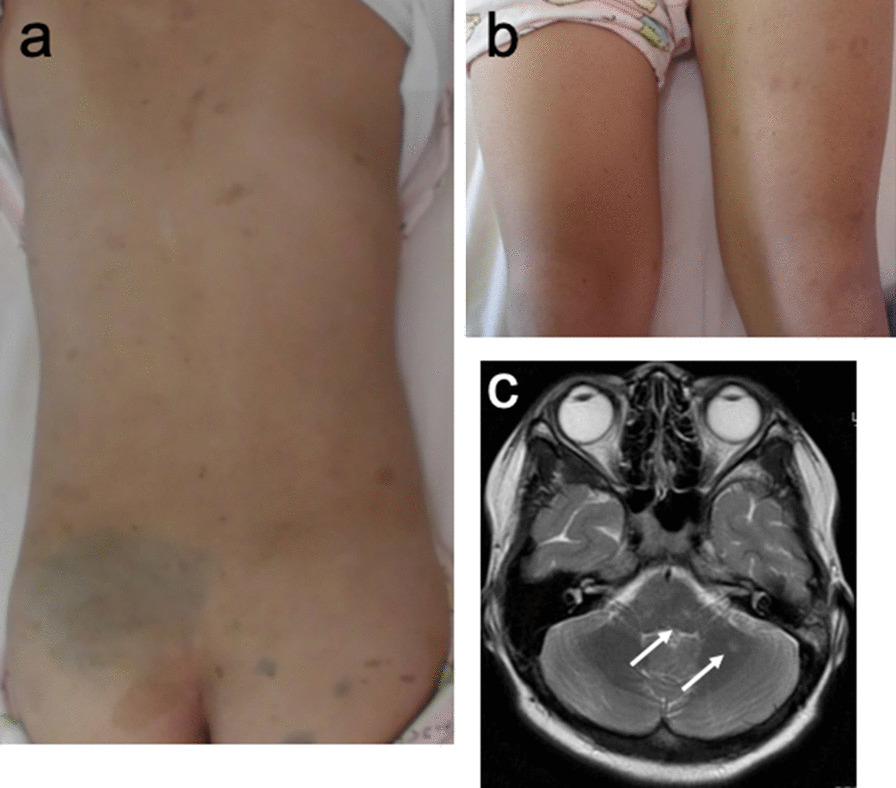
Café-au-lait macules (CALM) with irregular boundaries and uneven pigmentation on the dorsal area (**a**) and lower limbs (**b**). **c** Magnetic resonance imaging (MRI); arrows indicate unidentified bright objects (UBO) in the left cerebellar hemisphere

An 11-year-old girl exhibited persistent abdominal pain, diarrhea and bloody stool for two months. A bacterial infection was suspected and she was treated with antibiotics, but her symptoms persisted and she was referred to our hospital. Up to two years old, her medical history indicated she had undergone repeated ophthalmological examinations and brain magnetic resonance imaging (MRI) for suspected neurofibromatosis type 1 (NF1) because of multiple café-au-lait macules (CALM) present since birth. A family history showed her maternal grandmother had breast cancer in her 50 s, but no other history of CALM and/or colorectal cancer was recorded (Fig. 1). At admission, a physical examination revealed CALM on the dorsal area and the lower limbs (Fig. 2a, b). She had lost 3 kg in two months. Laboratory tests revealed mild anemia, and other blood tests were within normal limits. An ultrasonography demonstrated a mass protruding into the lumen of the descending colon. Colonoscopy (CS) revealed multiple polyps in the sigmoid and descending colon. The colonoscope could not pass the descending colon because the epithelial tumor occupied most of the colon lumen. Histology of the polyps revealed tubular adenoma (Group 3). Subsequent contrast-enhanced computed tomography showed a mass lesion in the ascending colon. A brain MRI showed faint T2-hyperintensities in the left cerebellar hemisphere, right pons, and right globus pallidus, indicating an unidentified bright object (UBO), potentially indicative of NF1 (Fig. 2c). No neoplastic lesions were observed.

Based on these endoscopic findings, it was highly likely the tumor in the descending colon was colorectal cancer. Approximately three weeks after admission, she was transferred to a university hospital to treat the colon polyps. After transfer, a second CS was successful for cecal intubation. This revealed multiple polyps in the transverse colon and ascending colon, and biopsy sampling revealed adenocarcinoma (tub1/tub2). One week later, polyps were removed by endoscopic mucosal resection and submucosal dissection. Pathological examination of four of the 19 polyps revealed moderately well differentiated tubular adenocarcinoma. Surgery was scheduled to remove the large tumor of the descending colon at a later date because it could not be removed endoscopically. Prior to surgery, the patient developed abdominal distension. MRI showed ascites and bilateral ovarian cysts with solid materials. Ascitic drainage was performed.

**Fig. 3:**
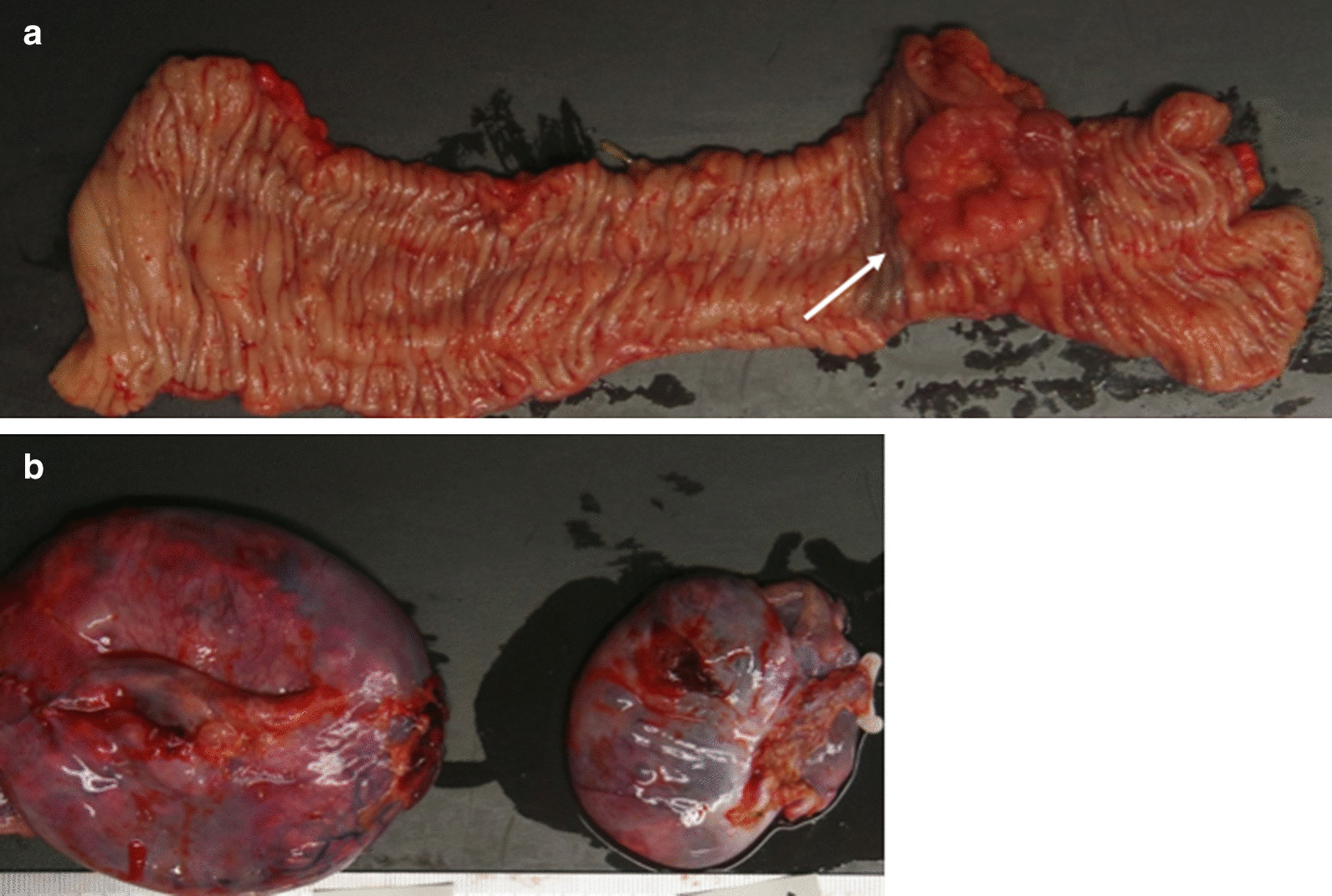
Macroscopic findings. **a** The arrow indicates the tumor in the descending colon. **b** The bilateral ovarian cysts with solid materials

Approximately four weeks after transfer, the patient underwent laparoscopic surgery. Bilateral ovariectomy was performed in addition to left hemicolectomy as adenocarcinomas were identified in ovarian solid materials, even though ascitic fluid cytology was negative for malignant cells (Fig. 3a, b).

Pathology of the resected colon and bilateral ovaries revealed adenocarcinoma. Histologically, it was difficult to determine whether the ovarian tumors were concurrent or metastatic from the primary tumor. The tumor of the descending colon had invaded from the muscularis propria to pericolorectal tissues. Lymph node metastasis was observed in one location (pT3N1aM1a, pStage IV).

**Fig. 4:**
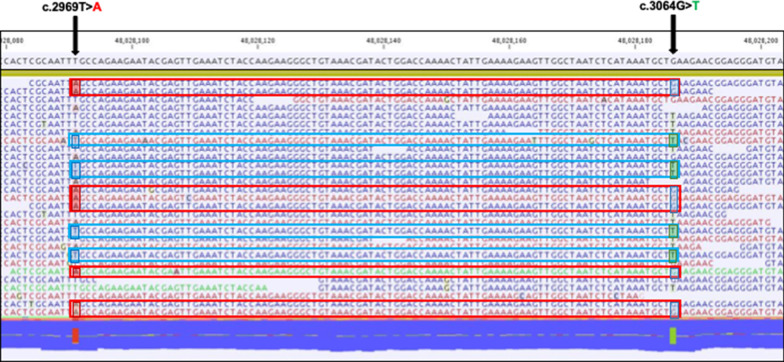
Biallelic variants in *MSH6* were identified using multi-gene analysis. Genetic testing revealed compound heterozygous variants; c. [2969T > A (p.Leu990*)] and [3064G > T (p.Glu1022*)] in *MSH6*

We also performed microsatellite instability (MSI) testing of genomic DNA from micro-dissected samples of the colorectal adenocarcinoma. This was conducted using the first panel of five microsatellite markers as recommended by the National Cancer Institute Consensus Conference [3]. The colorectal adenocarcinoma was classified as MSI-high (4/5). Immunohistochemical (IHC) tumor analysis indicated absent MSH2 and MSH6 protein expression, although MLH1 and PMS2 expression was positive. Taken together, CMMRD or Lynch syndrome (LS) was suspected. To be diagnostically definitive, we performed a multi-gene panel assay which contained 14 genes involved in hereditary colorectal cancer syndromes; *MLH1*, *MSH2*, *MSH6*, *PMS2*, *EPCAM*, *MSH3*, *MLH3*, *APC*, *MUTYH*, *POLD1*, *POLE*, *TP53*, *MBD4*, and *NTHL1.* The results revealed biallelic variants in *MSH6;* c.[2969 T > A (p.Leu990*)] and [3064G > T (p.Glu1022*)] (Figs. 1, 4). From this genetic information, the patient was diagnosed with CMMRD, indicating compound heterozygous variants in *MSH6*. This result suggested that loss of MSH2 protein expression in tumor tissue was probably due to secondary acquired somatic changes in *MSH2*. Parental and sibling genetic testing confirmed the patient was the biological daughter of her parents and an offspring of a non-consanguineous marriage. Her mother and sister had one variant of *MSH6*, c.3064G > T (p.Glu1022*), but the other variant, c.2969 T > A (p.Leu990*) was not identified in her parents, brother and sister. Her mother and sister who had the c.3064G > T variant never had LS-related tumors. These genetic analyses indicated the second variant, c.2969 T > A (p.Leu990*) had occurred de novo.

## Discussion and conclusions

We reported the case of CMMRD caused by two pathogenic variants in *MSH6*, one of which was de novo.

Childhood colorectal cancer (CRC) is extremely rare, with an incidence of approximately 1–3 cases per million in the United States [4]. Up to 2003, Yasuoka et al***.*** reported a total of 94 childhood CRC cases in Japan [5]. The pathogenic mechanism of childhood CRC is distinct to adults because it develops independent of lifestyle, with a very poor prognosis. Studies have reported that 10%–30% of childhood CRC cases are related to predisposing factors [6, 7]. These include familial adenomatous polyposis, ulcerative colitis, Crohn's disease, and Peutz-Jeghers syndrome [6]. Furthermore, limited case reports have shown that childhood CRC and adolescent LS, which is a hereditary cancer syndrome, are caused by a monoallelic pathogenic variant in one of the MMR genes [8–10]. Our case was suspected with a hereditary predisposition to cancer because she developed CRC at 11 years old. For this reason, she underwent genetic testing and was diagnosed with CMMRD.

CMMRD is a childhood cancer predisposition syndrome caused by biallelic homozygous or compound heterozygous pathogenic variants in one of the MMR genes [1]. The age at diagnosis of an initial CMMRD malignancy ranges between 0.4 and 39 years of age, with approximately 80% of cases occurring under 18 years old [11]. The tumor spectrum for CMMRD mainly includes hematological malignancies, brain/central nervous system tumors, LS-associated tumors, and other malignancies [2]. LS-associated tumors include colorectal, small bowel, endometrial, ureter, renal pelvis, biliary tract, stomach and bladder carcinoma [11]. The age of onset and tumor spectrum varies depending on the MMR genes [11].

Our case showed compound heterozygous pathogenic variants in *MSH6*; c.[2969 T > A (p.Leu990*)] and [3064G > T (p.Glu1022*)]. The c.3064G > T variant was present in her mother and sister. However, the c.2969 T > A variant was identified in neither parent. The patient also had a variant in *APC*, c.[6487A > C (p.Lys2163Gln)]. The allele frequency of this variant is 0.00002 according to the Genome Aggregation Database [12]. Her father also had the same rare APC variant, indicating she was her parent’s biological daughter.

Taken together, c.2969T > A was considered a de novo variant. Both c.3064G > T and c.2969T > A variants have previously been identified as pathogenic variants [13]. To the best of our knowledge, this is the first report indicating that one of the alleles in *MSH6* was a de novo variant in a pediatric CRC case, although a CMMRD patient with a de novo variant of the same gene, who developed B-cell acute lymphoblastic leukemia was previously reported [14]. Win et al*.* reported six de novo variant cases (in *MSH6*) in 261 LS patients [15]. Our investigation suggests that de novo variants in MMR genes are extremely rare in CMMRD.

CMMRD shows a wide range of clinical manifestations [11]. Patients display clinical features reminiscent of NF1 [16]. Multiple CALM was reported in 62% of CMMRD patients, and approximately 30% had other NF1 features (i.e., freckling, neurofibroma, lisch nodules, and UBO) [8]. It has been hypothesized that *NF1* is a frequent somatic target for MMR deficiency due to biallelic pathogenic variants in one of the MMR genes [17]. In support of this theory, studies have shown somatic *NF1* variants in MSI-high cell lines and MSI-high primary CRCs [17]. However, CALM in most CMMRD cases is < six occurrences; CALM in CMMRD exhibit irregular boundaries and uneven pigmentation, unlike the typical CALM in NF1 [8]. A recent study suggested that CALM in CMMRD could be distinguished from CALM in NF1 by experienced physicians, however some CMMRD cases exhibited typical NF1 CALM [16]. Furthermore, if an individual had atypical CALM, other CALM associated diseases (e.g., Legius syndrome, Noonan syndrome, and McCune-Albright syndrome) should be considered [18]. In our case, the patient showed UBO and atypical CALM. Since birth, she had undergone extensive tests for suspected NF1. She should have been screened for CMMRD because her CALM exhibited atypical features of NF1, e.g., irregular boundaries and uneven pigmentation. Suerink et al*.* reported that a 6-year-old girl without tumors was diagnosed with CMMRD [1]. Even though she met the diagnostic criteria for NF1, she did not exhibit *NF1* variants. Thus, she was suspected of having CMMRD, and genetic testing showed biallelic pathogenic variants in *PMS2*. In cases with NF1-like symptoms without NF1 variants or atypical CALM, CMMRD must be considered [19].

As CMMRD was first reported in 1999 [20, 21], further cases must be identified and investigated. Cases of suspicious NF1 or early childhood death from hematological malignancies or brain tumors may be undiagnosed for CMMRD. Going forward, it is important to inform physicians of CMMRD to allow patients receive appropriate clinical treatment and surveillance.

## Data Availability

Not applicable.

## Abbreviations

CMMRD: Constitutional mismatch repair deficiency
MMR: Mismatch repair
NF1: Neurofibromatosis type 1
CALM: Café-au-lait macules
CS: Colonoscopy
UBO: Unidentified bright object
MSI: Microsatellite instability
IHC: Immunohistochemical
CRC: Colorectal cancer
LS: Lynch syndrome
